# Reticulate Evolution of the Rock Lizards: Meiotic Chromosome Dynamics and Spermatogenesis in Diploid and Triploid Males of the Genus *Darevskia*

**DOI:** 10.3390/genes8060149

**Published:** 2017-05-24

**Authors:** Victor Spangenberg, Marine Arakelyan, Eduard Galoyan, Sergey Matveevsky, Ruzanna Petrosyan, Yuri Bogdanov, Felix Danielyan, Oxana Kolomiets

**Affiliations:** 1 Vavilov Institute of General Genetics, Russian Academy of Sciences, Moscow 119991, Russia; sergey8585@mail.ru (S.M.); yuri.bogdanov34@mail.ru (Y.B.); olkolomiets@mail.ru (O.K.); 2 Department of Zoology, Yerevan State University, Yerevan 0025, Armenia; arakelyanmarine@gmail.com (M.A.); petrosyan.ruzanna@mail.ru (R.P.); llacerta@ysu.am (F.D.); 3 Zoological Museum, Lomonosov Moscow State University, Moscow 125009, Russia; saxicola@mail.ru

**Keywords:** rock lizards, triploid, meiosis, synaptonemal complex, presynaptic alignment, hybridogenesis, DSB repair

## Abstract

Knowing whether triploid hybrids resulting from natural hybridization of parthenogenetic and bisexual species are fertile is crucial for understanding the mechanisms of reticulate evolution in rock lizards. Here, using males of the bisexual diploid rock lizard species *Darevskia raddei nairensis* and *Darevskia valentini* and a triploid hybrid male *Darevskia unisexualis × Darevskia valentini*, we performed karyotyping and comparative immunocytochemistry of chromosome synapsis and investigated the distribution of RAD51 and MLH1 foci in spread spermatocyte nuclei in meiotic prophase I. Three chromosome sets were found to occur in cell nuclei in the *D. unisexualis* × *D. valentini* hybrid, two originating from a parthenogenetic *D. unisexualis* female and one from the *D. valentini* male. Despite this distorted chromosome synapsis and incomplete double-strand breaks repair in meiotic prophase I, the number of mismatch repair foci in the triploid hybrid was enough to pass through both meiotic divisions. The defects in synapsis and repair did not arrest meiosis or spermatogenesis. Numerous abnormal mature spermatids were observed in the testes of the studied hybrid.

## 1. Introduction

The discovery of parthenogenetic reproduction in the rock lizards of genus *Darevskia* Arribas, 1997 [1,2,3] raised a fundamental biological question about the evolutionary mechanisms within complex taxons. The hybrid origin of unisexual species in rock lizards was an important proof of the role of reticulate evolution [4] in vertebrates generally, and in *Darevskia* genus in particular [5,6,7,8,9].

Further works demonstrated that hybridization is a common source of new evolutionary forms in tetrapods [10,11,12]. Hybrid forms arise as a result of the intersection between phylogenetic lineages in relatively short periods of time. Hence, the patterns of fusions of the close phylogenetic lineages resemble a network, rather than a tree [8].

Both diploidy and polyploidy occur in parthenogenetic reptile species, which have mechanisms to avoid the classical meiosis scenario. Clonal triploid species account for a major part of the known parthenogenetic reptile species [13] and are known in the genera *Leiolepis* [14,15], *Lepidodactylus* [16,17], *Hemidactylus* [16,18], *Heterontia* [19] and *Aspidoscelis* (syn. *Cnemidophorus*) [20,21,22]. 

Parthenogenetic species of genus *Darevskia* are diploids, which originate from hybridization between bisexual parental species [5,6,7,9,10]. The genetic structure of the genus *Darevskia* has been studied in detail [23,24,25]. However, many cytogenetic aspects of the observed phenomena remain unclear.

**Figure 1:**
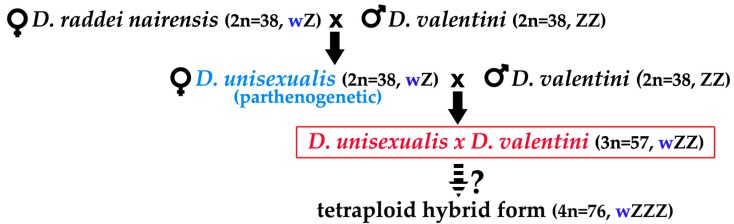
Hybridization scheme of natural triploid hybrids *Darevskia unisexualis* × *Darevskia valentini*. Bisexual diploid species *Darevskia raddei nairensis* and *Darevskia valentini* and diploid parthenogenetic species *Darevskia unisexualis*. Sex chromosome systems are indicated. The triploid hybrid studied in the current work was male. See text for details.

As has earlier been demonstrated by other authors [5,6,7,26,27], the diploid parthenogenetic species *Darevskia unisexualis* Darevsky, 1966 originates from the natural hybridization of two rock lizards, a *Darevskia raddei nairensis* Darevsky, 1967 female and a *Darevskia valentini* Boettger, 1892 male. *D. unisexualis* cells carry homeologous chromosome sets of the two parental species. Triploid hybrids are found in nature, in the sympatric populations of these species, resulting from natural matings between parthenogenetic *D. unisexualis* females and males of the bisexual species *D. valentini *(Figure 1).

Triploid rock lizard hybrids resulting from backcrosses of females from parthenogenetic *Darevskia* species with males from one of the parental species have been found to include sterile females and intersexes, as well as males and females with completely developed reproductive systems. Fertile triploid females have been considered to play a potential role in the origin of the tetraploid reptiles found in nature [22,26]. Tarkhnishvili et al. discuss the possibility of transition from triploid hybrids to diploid parthenogenetic forms [28]. Sterile triploid hybrid females have also been described in some species of the genus *Darevskia* [9,29,30], and in whiptail lizards (*Aspidoscelis dixoni C* × *A. tigris punctilinealis*) [31]. Triploid males of rock lizards often possess a completely developed reproductive system [9,26,32,33]. 

Karyotype analysis of hybrids in rock lizards revealed triploids (3n = 57, sex chromosome system wZZ), and a tetraploid specimen (3n = 76, sex chromosome system wZZZ), which combined features of bisexual paternal species *D. valentini* and unisexual species *D. unisexualis*, suggesting progressive steps of reticulate evolution for rock lizards and their potential for further evolution (Figure 1) [11,24,26,34,35].

Evolutionary roles for polyploid hybrids have recently been suggested for various taxonomic groups [17,22,36]. However, a key question in the theory of reticulate evolution is whether hybrids are fertile. Polyploids with an odd chromosome number usually have many imbalanced chromosomes and, consequently, are often sterile [24,37,38]. Triploids of a hybrid origin are not infrequent in reptiles. However, they often have reproductive problems due to the known limitations imposed by meiosis. 

Mature spermatids with atypical morphologies have been observed in the testes of hybrid triploid rock lizard males [9,38]. In addition, the spermatid count of hybrid triploid rock lizards has been found to be somewhat lower than in males of the parental species. Aneuploidy has been assumed for atypical spermatids [12,24]. Spermatogenesis proceeds to completion in such individuals, and germline cells pass through the so-called meiotic checkpoints in selection against abnormal spermatocytes. 

Thus, the question of whether viable gametes can be produced in triploid males, at least in minor amounts, is an important and interesting unsolved question in the biology of rock lizards. Viable gametes may result from recombination between chromosomes of the parental species. It is thought that crosses between triploid males and diploid females of a parental species may yield tetraploids with a restored normal meiosis. A similar scenario has been assumed for the origin of tetraploid lizards of the genus *Aspidoscelis* [33,39,40].

A detailed study of spermatogenesis in triploid hybrids is, therefore, important for understanding the mechanisms of reticulate evolution in rock lizard species [6,24]. 

The objectives of the present study were to compare chromosome synapsis, double strand break (DSB) repair, and meiotic recombination in meiotic prophase I of males of the bisexual rock lizard species *D. raddei nairensis* and *D. valentini* and a triploid hybrid male *D. unisexualis* × *D. valentini* as potential parents in the hybridization process, and to examine their spermatid morphology.

## 2. Materials and Methods

Three adult animals were captured and examined in May 2016 at the beginning of the breeding season and were deposited in the research collection of the Zoological Museum of Lomonosov Moscow State University (ZMMU). One male *D. raddei nairensis* (Amberd Castle population, ZMMU R-14956, specimen VS0003, collected by V. E. Spangenberg in May 2016), one male *D. valentini* (Mets Sepasar population, ZMMU R-14957, specimen VS0002, collected by V. E. Spangenberg in May 2016) and one hybrid male *D. unisexualis × D. valentini* (Kutchak population, hybrid zone, ZMMU R-14955, specimen VS0001, collected by V. E. Spangenberg in May 2016). Maintenance, handling and euthanasia of animals followed protocols approved by the Ethic Committee of the Institute of General Genetics. Experiments described in this manuscript were carried out in accordance with the approved national guidelines for the care and use of laboratory animals. Mitotic chromosomes were prepared from bone marrow and spleen following Ford and Hamerton [41] with modifications and fixed in an ice-cold acetic acid–methanol solution (1:3).

### 2.1. Synaptonemal Complex Analysis

Synaptonemal complex (SC) preparations were prepared and fixed using the technique of Navarro et al. [42] with modifications.

Antibodies and immunostaining procedure. Poly-L-lysine-coated slides were used for all immunofluorescence studies. The slides were washed with phosphate-buffered saline (PBS) and incubated overnight at 4 °C with primary antibodies diluted in antibody dilution buffer (ADB: 3% bovine serum albumin (BSA), 0.05% Triton X-100 in PBS) [43]. SCs were detected by rabbit polyclonal antibodies to the SC axial element protein SYCP3 (1:500–1:1000, Abcam, Cambridge, UK), DSB DNA repair sites were immunostained with mouse polyclonal antibodies to RAD51 protein (1:250–1:500, Abcam, Cambridge, UK), centromeres were detected by human anti-centromere antibodies CREST (1:500, Fitzgerald, Nort Acton, MA, USA), DNA mismatch repair protein MLH1 was immunostained with mouse monoclonal anti-MLH1 (1:250, Abcam, Cambridge, UK). After washing, we used the corresponding secondary antibodies diluted in ADB: goat anti mouse Immunoglobulin (Ig) G, Alexa Fluor 555 (1:500, Abcam, Cambridge, UK), Rodamine-conjugated chicken anti-rabbit IgG (1:400, Santa Cruz Biotechnology, Dallas, TX, USA), FITC-conjugated goat anti-rabbit IgG (1:500, Jackson ImmunoResearch, West Grove, PA, USA), goat anti-rabbit Alexa Fluor 488 (1:500, Invitrogen, Waltham, MA, USA), goat anti-human Alexa Fluor 546 (1:500, Invitrogen, Waltham, MA, USA). Secondary antibody incubations were performed in a humid chamber at 37 °C for 2h. Immunostaining was carried out sequentially in several rounds. The slides were examined using an Axioimager D1 microscope (Carl Zeiss, Jena, Germany) equipped with an Axiocam HRm CCD camera (Carl Zeiss, Jena, Germany), Carl Zeiss filter sets (FS01, FS38HE, and FS43HE) and image-processing AxioVision Release 4.6.3. software (Carl Zeiss, Jena, Germany). All preparations were mounted in Vectashield antifade mounting medium with DAPI (Vector Laboratories, Burlingame, CA, USA). 

### 2.2. Electron Microscopy

Slides for a subsequent electron-microscopic study were covered with Falcon plastic and contrasted with a 50% AgNO_3_ solution in a humid chamber for 3 h at 56 °C, then washed 4 times in distilled water and air dried. Then slides were examined under a light microscope to select mature spermatids. Once selected, Falcon plastic circles were cut out with a diamond knife, transferred onto grids, and examined at a JEM 1011 transmission electron microscope (JEOL Ltd., Tokyo, Japan).

The statistical significance of the difference of RAD51 and MLH1 foci number between parental species and the triploid hybrid was tested using the student’s t-test (two-tailed). The Origin Pro 9.0 software package (OriginLab Corp., Northampton, MA, USA) was used for descriptive statistics and diagram construction. When triploids and diploids were compared, the numbers of RAD51 foci were normalized to chromosome numbers. 

## 3. Results

### 3.1. Karyotyping in Males of the Parental Species D. raddei nairensis and D. valentini and Triploid Hybrid D. unisexualis × D. valentini 

**Figure 2:**
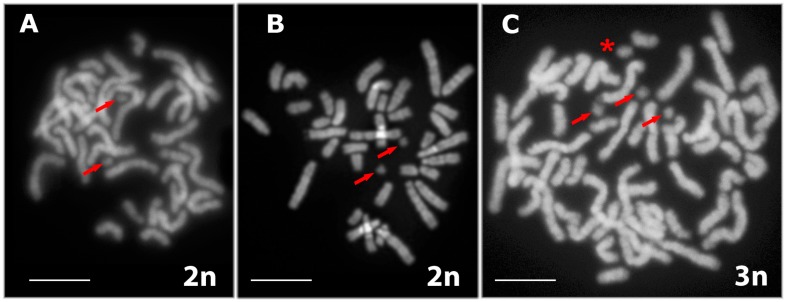
Metaphase plates of the lizard forms under study: (**A**) *D. raddei nairensis* with 2n = 38 (2n = 36A+ZZ); (**B**) *D. valentini* with 2n = 38 (36A+ZZ); and (**С**) *D. unisexualis* × *D. valentini* with 3n = 57 (54A+wZZ). Microchromosomes (m) are indicated with arrows, reduced type of the sex chromosome (w) is indicated with asterisk. Bar = 5 μm.

Karyotyping was carried out in rock lizards that had been captured in Armenia and represented the bisexual species *D. raddei nairensis* and *D. valentini*, as well as triploid hybrid *D. unisexualis* × *D. valentini* male. The triploid male had well-developed testes, of which one was larger than the other by approximately one-third. The male karyotypes were consistent with published data [24]: 2n = 38 (2n = 36A+ZZ) in *D. raddei nairensis*, 2n = 38 (36A+ZZ) in *D. valentini*, and 3n = 57 (54A+wZZ) in *D. unisexualis* × *D. valentini* (Figure 2). The reduced type of the w chromosome, inherited from maternal parental species (*D. raddei nairensis*) (Figure 1), was detected in the triploid individual karyotype (Figure 2).

### 3.2. Specifics of Chromosome Synapsis, DSB Repair, and Recombination in Males of the Parental Species D. raddei nairensis and D. valentini and in Triploid D. unisexualis × D. valentini Hybrid Male

#### 3.2.1. Specifics of Meiotic Prophase I in *D. raddei nairensis* Male

**Figure 3:**
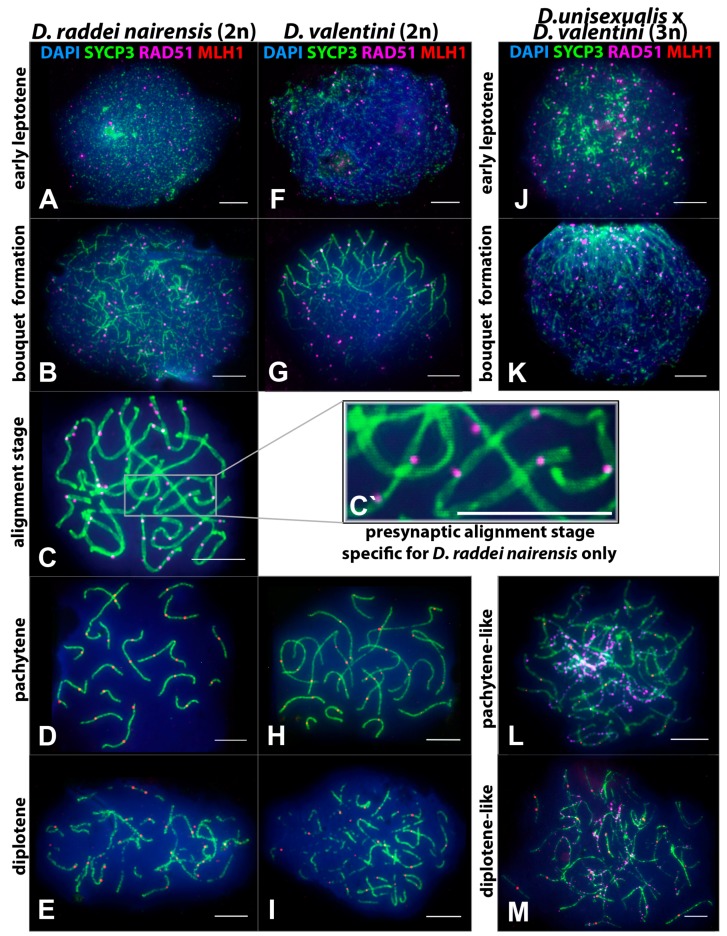
Preparations of total synaptonemal complexes (SC) from spermatocyte I nuclei in meiotic prophase I. Specifics of the chromosome synapsis, DNA double-strand breaks (DSB) repair and chromosome recombination in males of the two parental species (*D. raddei nairensis* and *D. valentini*) and in the triploid hybrid *D. unisexualis* × *D. valentini* male. Axial elements of the chromosomes and lateral elements of SC immunostained with antibodies against the SYCP3 protein (green). DNA DSB loci immunostained with antibodies against the RAD51 protein (magenta) and mismatch repair sites immunostained with antibodies against the MLH1 protein (red). Chromatin was stained with DAPI (blue). Bar = 5 μm.

Spread nuclei of spermatocytes I from male *D. raddei nairensis *(Figure 3A–E) were examined. Of all 165 prophase I nuclei, 3% were in early leptotene, 15.8% in zygotene, 19.4% in the alignment stage, 37% in pachytene, and 24.8% in diplotene stage (Table 1).

Classical leptotene, as a stage of long threads, was not detected in the *D. raddei nairensis* male. Assembly of axial elements from fragments and the start of homologous chromosome synapsis occurred simultaneously in different parts of the nuclei (Figure 3B). Chromosomes formed a bouquet formation, which is typical of many eukaryotes and is characterized by clustering of the telomeres of all chromosomes at one pole of the nucleus (Figure 3B). However, the bouquet formation was not followed by synapsis of homologous chromosomes and assembly of SC. A specific stage of homologous chromosome alignment was observed instead (Figure 3C,C**`**). Homologous chromosomes were coaligned but still showed no synapsis, and the distance between them was greater than the pachytene SC width. 

Intense DSB repair was observed as an appearing and then decreasing number of RAD51 foci from early prophase I to pachytene (Figure 3A–D). The remaining RAD51 foci were not eliminated at the alignment stage but remained associated with the axial elements of chromosomes at 48–61 per nucleus (Figure 3C). DSB repair was almost complete by pachytene when only several RAD51 foci were detectable. 

Pachytene in the *D. raddei nairensis* male was characterized by complete synapsis of all the chromosomes and the formation of 19 SC (18 autosomal bivalents and the ZZ sex chromosome bivalent). At this stage, MLH1 foci are detectable (mean of 24.29 ± 1.36 foci per nucleus) (Table 2, Figure 3D). It is clear that the MLH1 foci could not form yet at the alignment stage because homologous chromosomes did not contact each other. This is supported by an absence of MLH1 immunostaining (Figure 3C). 

Diplotene started with the formation of gaps in the SC structure (i.e., the SC began degrading before the start of chromosome desynapsis) (Figure 3D). 

#### 3.2.2. Specifics of Meiotic Prophase I in *D. valentini* Male

Spread nuclei of spermatocytes I from male *D. valentini* were examined (Figure 3F–I)*.* Of all 268 prophase I nuclei, 1.8% were in early leptotene, 23.5% in zygotene, 51.9% in pachytene, and 22.8% in diplotene stage (Table 1). 

A small number of early leptotene nuclei indicates that leptotene proceeds relatively fast in *D. valentini*. Chromosome axial structures assembly was often seen together with early bouquet formation (Figure 3G), but the alignment stage was not detected in any of the 268 nuclei examined.

The time course of DSB repair was generally the same as in *D. raddei nairensis* as inferred from the RAD51 focus number appearing and then decreasing with the progress of prophase I (Figure 3F–H). 

Pachytene was characterized by complete synapsis of 19 SC (18 autosomal bivalents and the ZZ sex SC bivalent) and the formation of MLH1 foci, which occurred at 29.12 ± 2.43 foci per nucleus on average (Table 2; Figure 3H).

Diplotene was similar to that observed in *D. raddei nairensis* in that the SC fragmentation preceded desynapsis of homologous chromosomes (Figure 3I).

- Thus, main characteristics and specifics of meiotic prophase I in males were established for *D. raddei nairensis* and *D. valentini*. A significant difference (*p* < 0.01) was found between the levels of meiotic recombination (number of MLH1 foci) of the two parental species (Figure 6B). We also detected a specific stage of homologous chromosome alignment, which was characteristic of the *D. raddei nairensis* male only (Figure 3C,C`).

#### 3.2.3. Specifics of Synapsis and DSB Repair in *D. unisexualis × D. valentini* Triploid Hybrid Male

Spread nuclei of spermatocytes I were examined in *D. unisexualis* × *D. valentini* triploid hybrid male, with 3n = 57 (Figure 3J–M). Of all 329 prophase I nuclei, 6.1% were in early leptotene, 26.7% in zygotene, 46.5% in a pachytene-like stage (see below), and 21.6% in the diplotene-like stage. The alignment stage was not observed (Table 1). 

The initiation of synapsis, the formation of a bouquet, and DSB repair in early prophase I were similar to those described above for males of the parental species, except that the chromosome number differed and that a larger number of RAD51 foci per nucleus was observed in early leptotene (Figure 3J,K). 

In the further prophase I stages, substantial differences in the dynamics of chromosome synapsis and DSB repair were observed in the hybrid compared with *D. raddei nairensis* and *D. valentini* males.

**Figure 4:**
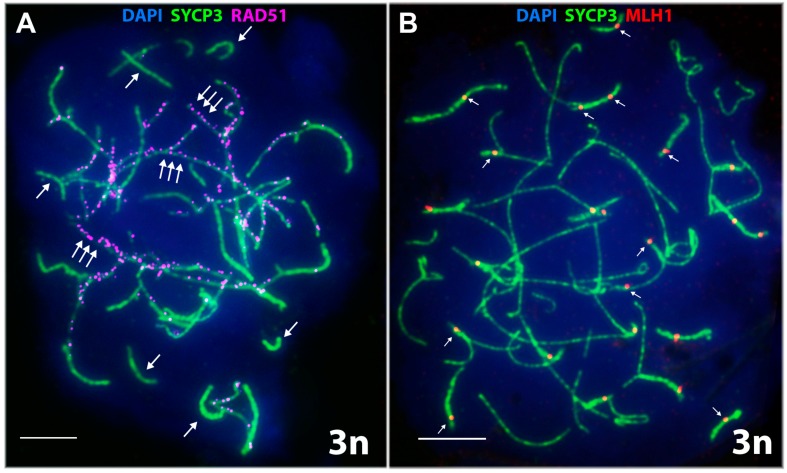
SC in spread spermatocyte I nuclei of a triploid *D. unisexualis × D. valentini* hybrid (3n = 57) immunostained with antibodies against the SYCP3 protein. (**A**) Late zygotene. Competitive synapsis of homeologous chromosomes. RAD51 foci (magenta) are numerous in long chromosomes (triple arrows) and absent in the short chromosomes (single arrows). (**В**) Early diplotene-like stage. Some of the MLH1 foci (red) are indicated by arrows. SC multivalents are seen. See text for comments. Bar = 5 μm.

**Figure 5:**
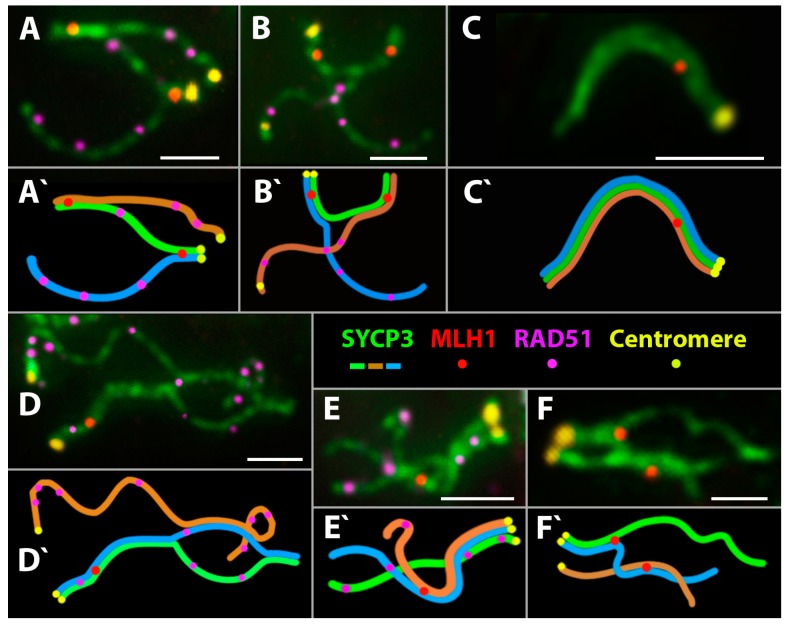
Specifics of competitive synapsis of homeologous chromosomes in spread nuclei of a hybrid *D. unisexualis × D. valentini* male (3n = 57) at the pachytene-like stage. (**A**–**F**) Axial elements of the chromosomes and lateral elements of SC immunostained with antibodies against the SYCP3 protein (green). DSB DNA repair sites immunostained with antibodies against the RAD51 protein (magenta), and mismatch repair sites immunostained with antibodies against the MLH1 protein (red). Centromeres immunostained with CREST antibodies (yellow). (**A`**–**F`**) Schemes of homeologous chromosome synapsis in SC trivalents. Homeologs in forming SC trivalents are shown with different colors. See text for comments. Bar = 2 μm.

A high frequency of nuclei with incomplete synapsis of homeologs was characteristic of meiotic prophase I in the triploid hybrid. Classical pachytene was not detected in any cell (Table 1). A prolonged process of homeologous synapsis yielded complex chromosome configurations, which were seen in the center of a spread nucleus (Figure 3L and Figure 4A,B). SC trivalents occurred rather often, forming from the axial elements of the three homeologous chromosomes involved in partial side-by-side synapsis. Synapsis was more common in pericentric or subtelomeric regions (Figure 4B and Figure 5A,B) and rare in interstitial regions (Figure 5D,E,F) of homeologous chromosomes. Full length univalents were also detected mostly in the center of a spread nucleus and were confirmed by numerous RAD51 foci (Figure 4A). The chromosomes were conventionally classified by length into long, medium and short.

**1.**  **Short chromosomes** (from 0.5 to 1 μm) formed SC throughout their lengths in nuclei from the triploid male. These SC already lacked RAD51 foci, but had one or, rarely, two MLH1 foci (Figure 3L and Figure 4A,B). Staining with anti-SYCP3 antibodies demonstrated that the short SC differed in width from SC bivalents observed in the diploid lizards. Electron microscopy is necessary to confirm or reject a trivalent nature of the thickened SC (Figure 5C).
**2.**  **Medium-length chromosomes** (from 1 to 3 μm) occurred more often at the periphery of a spread nucleus. These chromosomes showed side-by-side synapsis, or the SC formed only in the subtelomeric regions. However, synapsis of three medium-sized homeologs that was complete throughout the chromosome length was not observed. Likewise, RAD51 foci were not fully eliminated from the axial elements of chromosomes (Figure 3L, Figure 4А and Figure 5D,E).
**3.**  **Long chromosomes** (from 3 to 5 μm) often grouped in the central region of a spread nucleus. Synapsis of these chromosomes was usually incomplete, the finding being supported by arrays of RAD51 foci observed in asynaptic axis regions (Figure 3L and Figure 4А). Synaptic configurations of the long chromosomes were difficult to analyze without electron microscopy.

Thus, the pachytene-like stage was characterized by incomplete synapsis of homeologous chromosomes in SC trivalents (54 autosomes and the sex chromosomes w, Z, and Z) in the *D. unisexualis × D. valentini* male. 

The diplotene-like stage was identified by characteristic fragmentation of axial elements and desynapsis of homeologous chromosomes and was observed as often as in the parental species (Table 1). DSB repair was not completed at the diplotene-like stage, and only single nuclei had fewer than 50 RAD51 foci (Figure 3M). The number of RAD51 foci per nucleus mostly ranged from 157 to 51 at this stage (Figure 4A).

#### 3.2.4. Meiotic Recombination in Spermatocyte I Nuclei of the Triploid *D. unisexualis × D. valentini* Hybrid 

**Figure 6:**
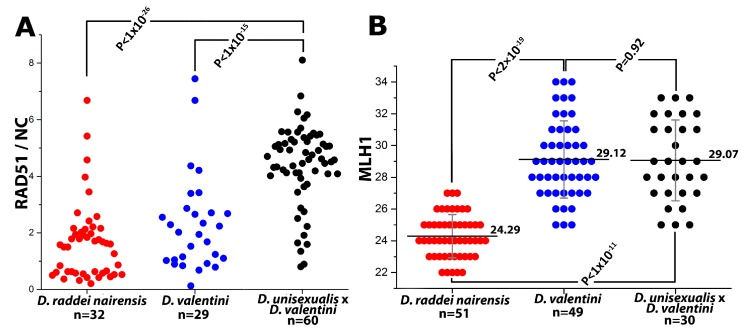
(**A**) Number of RAD51 foci per one chromosome (RAD51/NC, where NC indicates the chromosome number); and (**B**) Number of MLH1 foci per spermatocyte nucleus (mean ± SD) in *D. raddei nairensis, D. valentini*, and hybrid *D. unisexualis* × *D. valentini* male.

To our knowledge, this is the first reported observation of MLH1 foci in rock lizards. The MLH1 foci mark the sites of late recombination nodules (that is, prospective chiasmata) in regions of complete or incomplete homeologous chromosome synapsis in triploid spermatocyte I nuclei. On average, 29.07 ± 2.55 MLH1 foci per nucleus were detected in the triploid hybrid male (Table 2; Figure 4B and Figure 6B). 

One or two MLH1 foci per chromosome were detected in short and medium-length chromosomes with complete synapsis (Figure 4B). MLH1 foci were observed even in short synaptic regions of homeologous SC trivalents, including their subtelomeric, pericentric and interstitial regions (Figure 5А–F). On average, 1–2 MLH1 foci per medium-length SC trivalent were observed (Figure 5A–F). Immunogold staining and electron microscopy are now needed to study the specifics of synapsis in the central areas of spread nuclei.

### 3.3. Comparative Study of Mature Spermatids of D. raddei nairensis and D. valentini and Hybrid D. unisexualis × D. valentini Male 

**Figure 7:**
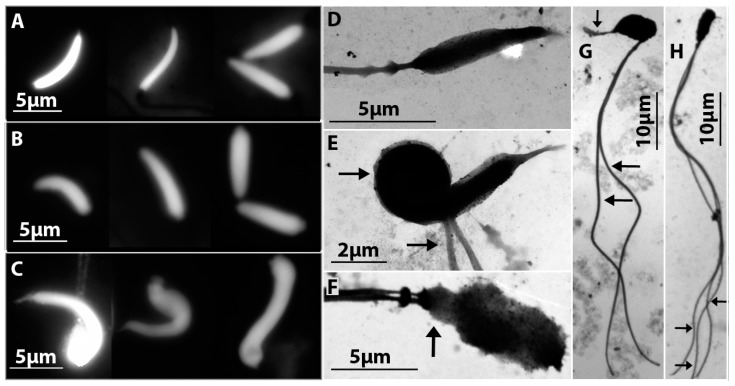
(**A**–**C**) spermatid heads, DAPI staining: (**A**) *D. raddei nairensis*; (**B**) *D. valentini*; and (**C**) *D. unisexualis × D. valentini. (***D**–**H**) electron microscopy of spermatids, silver nitrate staining: (**D**) *D. valentini*; and (**E**–**H**)* D. unisexualis × D. valentini*. A presumably aneuploid spermatid (**E**) has a swelling at the head base and two flagella. Spermatid abnormalities (**F**–**H**) included a heteroaxial pattern, two or three flagella, and an enlarged or deformed head. The abnormalities are indicated with arrows.

Mature spermatids were observed by fluorescent and electron microscopy of spread spermatocyte I nuclei from diploid and triploid lizards. In *D. raddei nairensis* and * D. valentini* males, spermatids displayed features characteristic of the species, including an elongate head, a uniform chromatin staining, and always a single flagellum (Figure 7А,B,D), and generally corresponded to descriptions of reptile sperms [44,45,46].

Several morphological abnormalities were observed for spermatids of the triploid *D. unisexualis × D. valentini* hybrid (Figure 7C,E–H). For instance, many spermatids were heteroaxial (the angle between the head and flagellum <180°), while others had an enlarged, swollen, deformed, decreased, or totally reduced head. Spermatids with two or three flagella and structural abnormalities of the acrosome and neck region were observed (Figure 7E–H). No normal spermatid (morphologically similar to spermatids of parental species) was detected in any of the 281 spermatids examined in the *D. unisexualis × D. valentini* male.

## 4. Discussion

### 4.1. Meiosis in Bisexual Parental Species D. raddei nairensis and D. valentini Males

In total, events of meiotic prophase I in *D. raddei nairensis *(2n = 38, ZZ) and *D. valentini* (2n = 38, ZZ) males follows the classical scenario characteristic of the homogametic sex in animals. This concerns the time course of the formation of chromosome axial elements, the organization of a bouquet formation, and the time course of DSB formation and repair.

The most significant finding is that the average number of MLH1 foci (prospective chiasma sites) per nucleus differs between *D. raddei nairensis *(24.29) and *D. valentini* (29.12) suggests different levels of meiotic recombination for the species (Figure 6B).

Another significant distinction between two parental species studied is a specific stage of homologous chromosome alignment that occurs in early prophase I in *D. raddei nairensis *(Figure 3С–C**`**). Situations where homologs are brought close together prior to synapsis have been described for several plants and fungi (cited from [47]). In animals, a presynaptic alignment of homologs has been observed in the locust *Locusta migratoria* [48]. Studies have shown that homologous chromosomes are coaligned and are approximately 400 nm (300 nm in some studies) apart at this stage and that the distance between them decreases to 100 nm after elongation of SC assembly [47]. Alignment of homologous chromosomes prior to synapsis has been associated with the formation of their common separate domains, which prevent a trapping of non-homologous chromosomes [47].

The presence of RAD51 foci (often paired) at the alignment stage in axial elements brought close together (Figure 3C–C**`**) indicate that DSB repair is incomplete at this stage and associate DSB repair with the initiation of homologous chromosome synapsis. The recognition and alignment events seem to be synchronized for all bivalents prior to a transition to the next stage of meiotic prophase I, that is, SC assembly.

Pachytene strictly follows the classical scenario in both *D. raddei nairensis *(Figure 3D) and *D. valentini *(Figure 3H). The telomeric regions of bivalents are regularly spread through the nuclear membrane, and homologous chromosome synapsis is complete at this stage.

Fragmentation of the axial structures of bivalents marks the start of diplotene and is followed by lateral element desynapsis in males of the two species (Figure 3E,I). This order of diplotene events has been observed before in mice, humans and several other species [49]. In both of the parental species, males produce numerous spermatids that are typical of *Lacertidae* and have elongate fusiform heads (Figure 7A,B).

### 4.2. Meiotic Recombination in Spermatocytes of Triploid D. unisexualis × D. valentini Male 

To produce viable sperm, germline cells must successfully pass through meiosis and, first of all, through meiotic prophase I, which has a number of checkpoints. The checkpoints act to ensure strong selection, in particular, against spermatocytes with incomplete synapsis, incomplete DSB repair, or distorted chromosome desynapsis [50].

Analyzing meiotic prophase I in the triploid, we observed delayed DSB repair apparently associated with extended asynaptic regions seen in the triploid nucleus. RAD51 foci mark well the unpaired axes of homeologous chromosomes (Figure 3L,M and Figure 4A). Three homeologous chromosomes of the triploid hybrid *D. unisexualis × D. valentini* male show only partial synapsis and form intricate synaptic configurations in spermatocyte I nuclei. The configurations are not always possible to decipher because the asynaptic axial elements are often stretched to a great extent, interlocked, or involved in temporary associations (Figure 4A,B).

Resuming our cytological observations, we found that the process of homologous and homeologous chromosome synapsis compete in spematocytes of the triploid male under study. Not a single spermatocyte I nucleus with complete chromosome synapsis was detected. It is known that, in mammals, such defects in synapsis trigger a checkpoint that removes cells via apoptosis. 

However, MLH1 foci were observed in regions of SC assembly (Figure 3L–M, Figure 4B and Figure 5A–F). The finding suggests a successful formation of chiasmata.

We found MLH1 foci not only between homologs, but between homeologs as well (Figure 5A,B,F). In general, the finding means that, after more or less successful prophase I, spermatocytes of the triploid animal can enter meiotic division. On the other hand, there is obvious lack of the number of chiasmata detected in triploid nuclei. Assuming a minimum of two chiasmata per trivalent, there should be 38 MLH1 foci rather than 29 detected per nucleus on average (Figure 6B). Actually, we detected two MLH1 foci only for 3–6 trivalents per nucleus and full length univalents with no MLH1 foci were described as well (see Section 3.2.3 in Results). 

We did not study the MI and MII preparations. However, numerous spermatids have been detected in triploid hybrid. This clearly shows that spermatogenesis has not been blocked in mid pachytene and that cells have passed through meiotic divisions.

All 281 spermatids examined had morphological abnormalities (Figure 7C,E–H) (see Section 3.3 in Results for details) [9]. Resolution of the complex synaptic structures in meiotic anaphase I apparently makes it highly likely that sperms with aneuploid nuclei are produced in the *D. unisexualis* × *D. valentini* hybrid [12,24,51]. It is not surprising as soon as, theoretically, meiosis has to be aberrant in hybrid triploids, even if chiasmata form between homeologous chromosomes.

It is reasonable to predict that aneuploid spermatozoa are incapable of fertilizing an egg in natural conditions [52,53,54,55,56]. However, a unique finding of a tetraploid hybrid male (4n = 76, wZZZ) was made in April 2004 in the Kuchak hybrid zone. The lizard was similar in coloration and pholidosis to triploid hybrid males, and numerous spermatids and spermatozoa were detected. The origin of the tetraploid male is unclear, but its mere existence was assumed to provide evidence for partial fertility of either female or male triploid hybrids *D. unisexualis* × *D. valentini* (Figure 1) [26].

Triploid hybrids have been found at a high frequency in hybrid zones [26]. The number of hybrids that appear annually in this sympatric zone is extremely high and exceeds 33% of the mixed population. The portion of hybrid individuals in other mixed populations does not exceed 7–12% [11]. All these data mean that there is a permanent source of carriers of ovules and sperms in the mixed population, maintaining the pool of hybrid animals in this hybrid zone. Thus, the question of the origin of tetraploid individual is still open. Laboratory crosses should be performed to check this assumption. Such studies are of importance for verifying Darevsky’s hypothesis that triploid males are involved in a transition to tetraploidy [26].

### 4.3. Comparison of Our Findings with Previous Observations 

Note in this respect that many spermatogenetic defects of meiosis and premeiotic stages have earlier been observed with the use of light microscopy in triploid male Armenian rock lizards similar to the triploid studied in this work [24,26]. Light microscopy has detected signs of distorted chromosome synapsis in the hybrids, but the questions about the formation, number, and localization of chiasmata have not been resolved. Rare spermatids have been found in triploid males [24]. As for the production of mature spermatozoa, the available data are discrepant. Sperms have not been detected in some studies [24], but have been detectable and found to be abnormal [57] or apparently normal in other work [26]. 

### 4.4. Is Morphology of Spermatids or Spermatozoa Indicative of Fertility or Infertility? What Is Necessary Bridging Cytogenetics of Spermatogenesis with Reticular Evolution? 

Interconnections of hybridization, parthenogenesis, and polyploidy provide a basis for the hypothesis of a reticular origin of species [8,58]. Reticular evolution suggests that new taxa originate via combinations of already existing ones.

Diploid parthenogenetic lizards resulting from interspecific hybridization are capable of hybridizing with the parental species to produce triploids and even tetraploids [26]. Fertility of hybrids is a key issue in the problem of reticular evolution. We have not found out whether triploid *D. unisexualis* × *D. valentini* males are fertile. The hybrid we examined had well developed testes, and the spermatocytes passed through all checkpoints of the first and second meiotic divisions and produced numerous morphologically abnormal spermatids (Figure 7C,E–H). Are all of these spermatids infertile, or are some of them fertile? We do not now yet. Alterations in morphology of spermatozoa do not necessarily suggest infertility, as well as a normal spermatozoon structure does not necessarily suggest fertility, for instance, in human [59,60].

## 5. Conclusions

To summarize, a cytogenetic study of meiotic prophase I was carried out in a triploid *D. unisexualis × D. valentini* male and diploid males of the parental species (*D. raddei nairensis* and *D. valentini*). We found that spermatocytes of the triploid hybrid pass through meiotic prophase I, as well as through both meiotic divisions. We demonstrate that defects in chromosome synapsis and prolonged incomplete DSB repair do not block meiosis in the triploid *D. unisexualis × D. valentini* hybrid male. The evidence of crossing over in trivalents of homeologous chromosomes was obtained in *D. unisexualis* × *D. valentini* male. Numerous abnormal mature spermatids were observed in the testes of the studied hybrid, but the observation neither proves nor disproves fertility of tripoid animals and their possible participation in further reproduction to yield tetraploids. Laboratory breeding experiments of lizards are necessary for testing the probability of occasional fertility of the triploids and if they may be involved in events of reticular evolution.

## Figures and Tables

**Table 1:** Distribution of spread spermatocyte I nuclei through the stages of meiotic prophase I in the parental species (*D. raddei nairensis* and *D. valentini*) and triploid *D. unisexualis × D. valentini* hybrid male. See text for comments.

Species	Early Leptotene	Zygotene	Alignment	Pachytene	Diplotene	Total Nuclei
***D. raddei nairensis ***	5 (3%)	26 (15.8%)	32 (19.4%)	61 (37%)	41 (24.8%)	165
***D. valentini***	5 (1.8%)	63 (23.5%)	0	139 (51.9%)	61 (22.8%)	268
***D. valentini × D. unisexualis***	20 (6.1%)	88 (26.7%)	0	150 (45.6%) ^1^	71 (21.6%) ^2^	329

^1^ A pachytene-like stage was observed (i.e., classical pachytene was absent) and complete synapsis of homeologous chromosomes was not observed in any cell; ^2^ A diplotene-like stage was observed; elimination of RAD51 foci was not observed. Degradation of the synaptonemal complex, desynapsis, and MLH1 foci were detected.

**Table 2:** Karyotype and the number of MLH1 foci per spermatocyte I nucleus (mean ± SD) *D. raddei nairensis*, *D. valentini* and the hybrid *D. unisexualis* × *D. valentini* male.

Species	Karyotype	*N*	MLH1 Focus Number Per Nucleus, Mean ± SD
*D. raddei nairensis*	2n = 38	51	24.29 ± 1.36
*D. valentini *	2n = 38	49	29.12 ± 2.43
*D. valentini* × *D. unisexualis*	3n = 57	30	29.07 ± 2.55

*N* is the number of spermatocyte nuclei examined, SD is Standard Deviation.
